# Management of periprosthetic fractures around cemented femoral stems: osteosynthesis and revision strategies

**DOI:** 10.1530/EOR-2024-0205

**Published:** 2026-06-01

**Authors:** Jeffrey W Kwong, Cary S Politzer, Stefano A Bini, Paul Toogood, Claudio Diaz-Ledezma

**Affiliations:** Department of Orthopaedic Surgery, University of California San Francisco, San Francisco, California, USA

**Keywords:** periprosthetic femur fracture, periprosthetic fracture, femur fracture, cement, cemented stem, revision arthroplasty, open reduction internal fixation, osteosynthesis

## Abstract

- Periprosthetic femur fractures most often occur in elderly, frail patients and represent a devastating injury with high rates of morbidity and mortality even with appropriate surgical management.
- Hip arthroplasties with cemented femoral fixation have a decreased risk of periprosthetic fracture relative to cementless designs, especially when composite beam stems are used compared to polished, taper-slip stems.
- Early mobilization is the primary goal of treatment for periprosthetic femur fractures, which can be achieved either through open reduction internal fixation or through revision arthroplasty.
- Where the pattern is reconstructable, such as in most B1 patterns and some B2 fractures around polished, taper-slip stems, open reduction internal fixation of fractures around cemented stems has been associated with decreased perioperative complications compared to revision arthroplasty.
- In the setting of a compromised bone–cement interface, inadequate metaphyseal bone stock, or implant breakage, revision arthroplasty is the preferred treatment strategy.

## Introduction

Periprosthetic femur fractures (PFFs) represent a severe complication following total hip arthroplasty (THA) and hemiarthroplasty (HA), and treatment of periprosthetic fractures around a cemented stem (CS) can be complex. There has been a recent resurgence in the use of cemented femoral stems in both elective hip replacement and surgery for hip fractures, particularly in patients who may be at a higher risk of periprosthetic fractures with uncemented fixation. Surgical management requires combining advanced concepts in adult reconstruction and orthopedic trauma together with a thorough evaluation of the patient’s needs and expectations. The aim of this review is to add perspective into the management of periprosthetic hip fractures specifically involving cemented femoral stems, which have unique considerations compared to fractures around stems with uncemented fixation. We hope to provide insight and technical tips into when and how to use osteosynthesis versus revision arthroplasty in the treatment of these complex fractures.

## Epidemiology

### Risk of periprosthetic fractures in cemented stems after total hip arthroplasty and after hip fractures

According to a systematic review (1), PFFs may be observed intraoperatively in 0.1–8.2% of cases. Postoperatively, they occur at a rate of 0.07–3.5%. The lifetime risk of revision due to periprosthetic fractures was studied in the New Zealand Joint Registry and ranges from less than 1% in women 81–85 years old to 5.3% in men 45–50 years old (2). Males had twice the rate of revision due to periprosthetic fractures compared to females across all age categories. Osteoporosis is also a well-known risk factor for PFFs, although the exact prevalence and relative increase in risk of fracture due to osteoporosis have not been well characterized (3, 4).

While cemented femoral fixation has been associated with a 7- to 20-fold lower odds of PFFs compared to cementless fixation (5, 6, 7), fractures with CSs nonetheless occur at an estimated rate of 0.1–0.5% (5, 8). Baryeh *et al.* described a 1.5% risk of PFFs in patients with CSs, with an average time from primary operation of 71 months. These fractures mainly occur after low-energy falls (1).

In the setting of THA for femoral neck fractures, the risk of revision due to PFFs observed with CSs is ten times lower compared to uncemented stems (0.07 vs 0.74% at 30 days), with no reported revisions at 90 and 180 days for CSs in 4,427 THAs (6).

### Cemented stem designs and risk of fracture: not all cemented stems are equal

Observational evidence suggests the risk of PFFs is influenced by the design of the CS. Two categories (9) include composite beam (CB) stems, which provide rigid fixation between the stem, cement, and bone by using large surface areas and textured surfaces, and polished, taper-slip (PTS) stems, which allow gradual, limited subsidence within the cement mantle (10, 11). Multiple studies, including registry data from Sweden and the United Kingdom, demonstrated that the risk of PFFs with PTS stems is 10–16 times greater compared to CB designs (5, 11, 12, 13, 14, 15). A proposed explanation is that the controlled subsidence of the PTS stem results in a stress riser, resulting in a wedge effect that splits the femur after a direct hip contusion (11, 16). Certain PTS stem designs have also been associated with a higher risk of PFFs. For example, the Zimmer Biomet CPT stem, which has now been phased out, was found to have higher stress at the critical point of failure, resulting in fractures at lower torques (17). In addition, most PFFs around CB stems occur later (15). Recent evidence suggests that a collared design may be protective against PFFs (18). Anatomic stems have also been associated with lower rates of PFFs, perhaps because their anatomic design facilitates better alignment and more distal anchoring, resulting in greater resistance to torque forces (11, 15, 19).

## Diagnostic challenges

### The Vancouver classification

The fracture pattern observed with CSs may be distinct from that of cementless stems. While spiral, ‘sickle-like’ fracture patterns are commonly seen with both cemented and cementless stems, PTS stems can also result in a comminuted ‘axe-splitting’ pattern (10, 20).

The Vancouver classification guides the treatment of PFFs. It considers the location of the fracture, stability of the stem, and quality of the bone (21). However, the Vancouver classification does not consider patient factors, such as functional requirements and medical comorbidities. Consequently, while the classic treatment algorithm of the Vancouver classification may apply for most patients, some exceptions can be found in clinical practice.

Vancouver A fractures involve the trochanters. A_G_ involves the greater trochanter, and A_L_ involves the lesser trochanter. Vancouver B fractures occur around the femoral stem or slightly distal to its tip. In B1 fractures, the stem is stable. In B2 and B3 fractures, the femoral component is unstable. B2 fractures have adequate bone stock about the implant, and B3 fractures have inadequate bone stock. Vancouver C fractures occur distal to the tip of the implant. Prior reports have found that PTS stems most commonly have PFFs with a B2 pattern (39.5%) (1).

**Figure 1:**
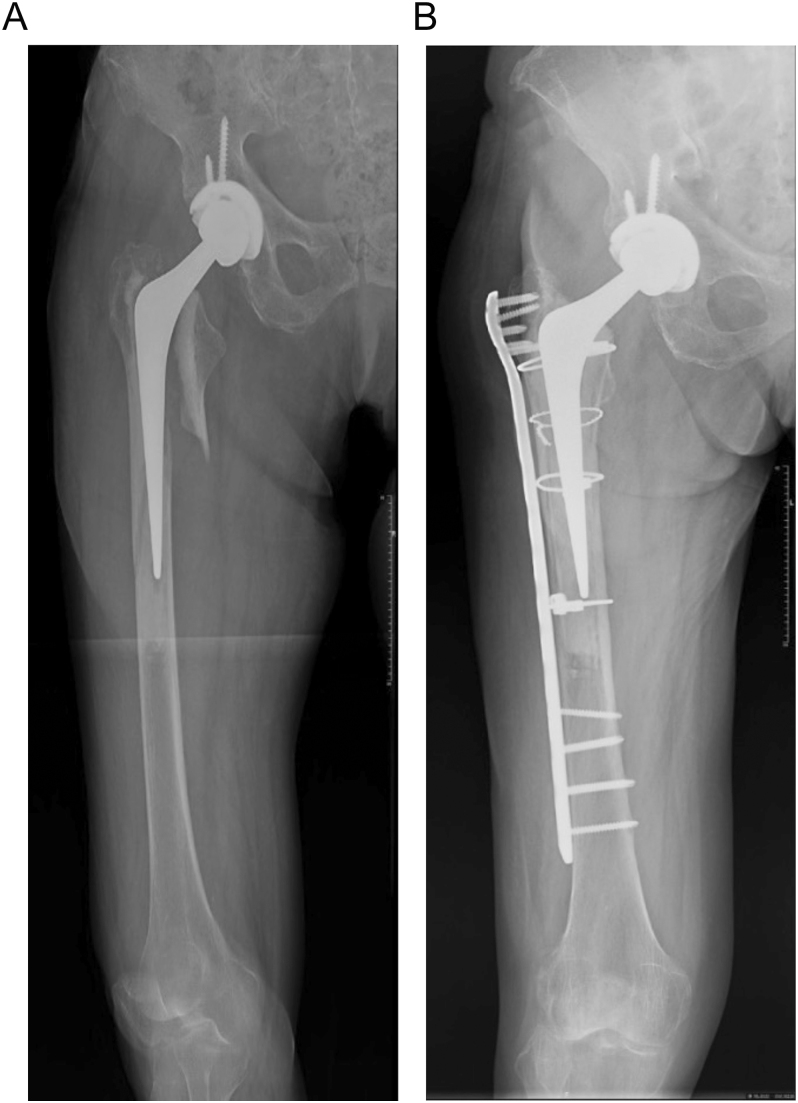
(A) Vancouver B2W involving a polished, taper-slip stem. (B) ORIF involving cerclages and a locking plate for a B2W fracture.

**Figure 2:**
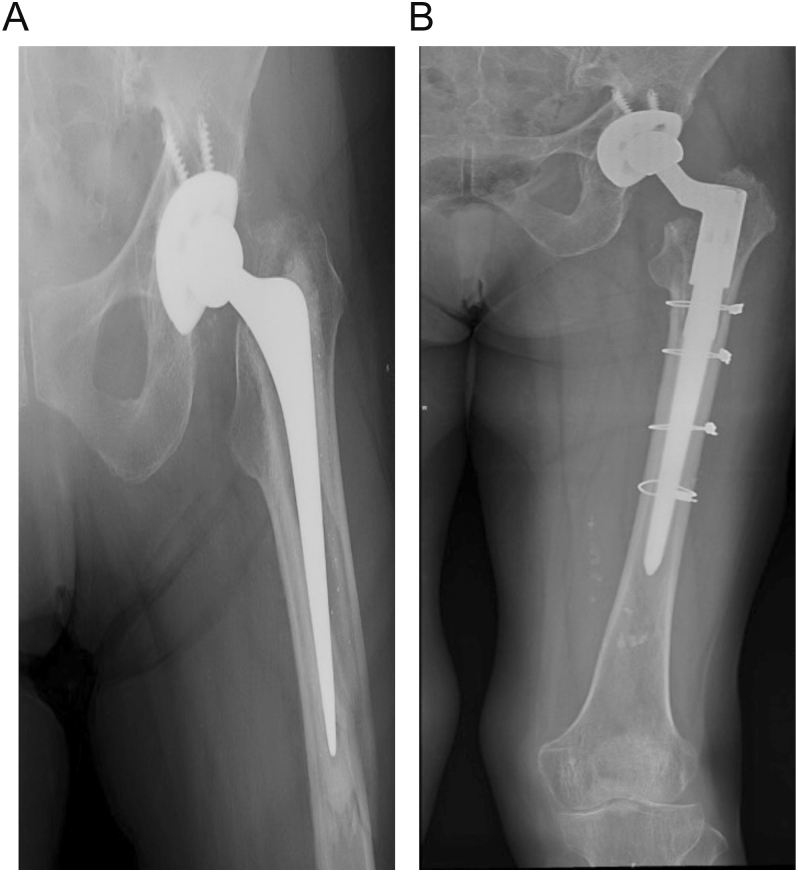
(A) Vancouver B2L involving a polished, taper-slip stem. (B) Uncemented, fluted, modular, tapered stem revision of a B2L fracture.

Maggs *et al.* (22) modified the Vancouver classification to further delineate B2 patterns specifically in CSs. B2W describes PFFs where the cement is well fixed to the bone (Fig. 1A), and B2L describes cases where the cement is loose from the bone (Fig. 2A). The authors reported that B2W patterns are more frequently seen in PTS, and B2L, in CB designs.

While the Vancouver system remains the most widely used classification system of PFFs, it does not fully capture the nuances of all fracture patterns, especially for complex cases. For example, the Vancouver system does not account for the fracture pattern, which can be important in guiding treatment. It has also been reported to have inadequate validity for B type fractures around PTS stems (23). We advocate for the routine use of CT scans when evaluating PFFs to understand the anatomy of the fracture in greater detail and to understand the relationship between the implant and the surrounding bone and cement mantle, as is further discussed (expert consensus). For example, CT scans can identify occult, minimally displaced diaphyseal extension of the fracture and thus influence the decision for the length of fixation, and they can help identify significant disruption of the bone–cement interface that may sway the decision to perform revision arthroplasty over osteosynthesis.

The unified classification system (UCS) was developed in 2014 to build upon the Vancouver classification system. Designed to allow greater generalizability, the UCS can be applied to periprosthetic fractures of any joint, not just for fractures around the hip. While type A, B, and C fractures in the UCS are essentially the same between the Vancouver system and the UCS, the UCS has several additional categories to describe other periprosthetic fracture scenarios. This includes type D, which describes interprosthetic fractures between replacements of two different joints, type E, which involves fractures of each bone supporting a single replacement, and type F, which involves fractures of a native joint surface articulating with an implant, such as an acetabular fracture in the setting of a hemiarthroplasty of a hip (24).

Of note, UCS type D fractures between a THA and a total knee arthroplasty are increasingly common and present unique treatment challenges. Specific strategies are needed to address the decreased bone stock and issues at both the hip and the knee. While the treatment of type D fractures are beyond the scope of this review, we strongly recommend that patients with a PFF who have a history of both a prosthetic hip and a prosthetic knee receive adequate imaging to rule out the possibility of an interprosthetic fracture, including full-length femur radiographs and CT scans covering the entire femur (expert consensus).

To date, however, the Vancouver classification system remains the dominant system for describing PFFs, perhaps because of familiarity from its historical use or because the expanded categories of the UCS (i.e., types D, E, and F) are relatively less common (25).

## Treatment

### Bone–cement and cement–implant interfaces

When addressing PFFs in a CS, the evaluation of the cement mantle is important. Two interfaces exist: the bone–cement interface and the cement–implant interface. According to the Harris criteria (26), a broken cement mantle is indicative of loosening and immediately classifies the PFF as Vancouver B2, requiring revision surgery. However, a recent study by Smitham *et al.* (27) challenged this concept. The authors noted that if the bone–cement interface is adequate, open reduction and internal fixation (ORIF) using the same stem can effectively address the PFF (Fig. 1B). Two later publications (10, 28) reinforced the observations of Smitham *et al.* Another series from Argentina (29) demonstrated that careful selection of patients with a B2 pattern for osteosynthesis (frail and low-demand patients with polished CSs) can be associated with good results, comparable to those observed in B1 patterns. Consequently, not all Vancouver B2 fractures require revision of the femoral stem. However, ORIF is only viable if an anatomical reduction of the bone and cement mantle is achieved.

**Figure 3:**
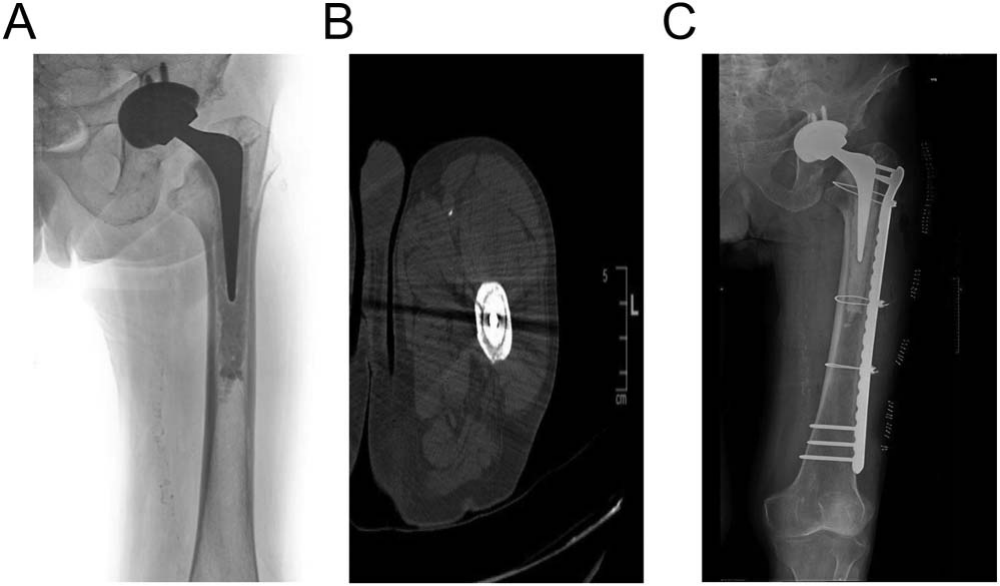
(A) ‘Lucent line sign’ observed in Gruen zone 7, with no other radiographic findings of fracture, in an 86-year-old patient with a low-energy fall and hip pain evaluated at the emergency room. (B) Axial CT scan image confirming a PFF. (C) Postoperative image after ORIF, showing disappearance of the ‘lucent line sign’ at Gruen zone 7.

The bone–cement interface should be evaluated on preoperative images, and its integrity should be confirmed during surgery. Scrutinizing radiographs prior to the fracture is also helpful. If pre-fracture X-rays show loosening at the bone–implant interface, the PFF should be considered a B2L. Maggs *et al.* also described the ‘lucent line sign’, which is indicative of a minimally displaced, often missed, PFF around a PTS stem (Fig. 3A, B, C) (30).

### Periprosthetic joint infection (PJI) and osteolysis

A recent publication covers the topic of PFF and PJI in depth (31) and suggests that PJI may be a concomitant diagnosis in over one in ten cases. In this situation, femoral implant loosening can occur due to the fracture with subsequent infection, or osteolytic infection and chronic inflammation can result in a progressively loose femoral implant, resulting in fracture (32). Therefore, PJI should be considered in all cases of PFFs. Trauma can result in elevated serum and local inflammatory response regardless of infection. van den Keiboom *et al.* found that the Musculoskeletal Infection Society thresholds for PJI in the setting of trauma have high sensitivity but low specificity, and they advocated for higher threshold values for patients with PFFs (33). However, they and others have not identified specific cutoffs for identifying PJI in the setting of PFFs, citing the need for further studies (31). Thus, the diagnostic course should be tailored according to pretest probability.

For all patients with PFFs, we recommend evaluating serum C-reactive protein (CRP) and erythrocyte sedimentation rate (ESR). However, in cases of low pretest probability for PJI, these results should not delay surgery but can be used in combination with intraoperative synovial fluid and tissue culture results. In cases of high preoperative clinical suspicion for PJI (the patient reports hip pain prior to the fracture, and loosening or osteolysis likely contributes to the fracture), serum CRP and ESR can guide a diagnostic workup. If both are negative, then infection can be ruled out. If one or both are positive, then joint aspiration should be performed to evaluate synovial fluid white blood cell count and polymorphonuclear percentage (PMN%). The utility of intraoperative frozen section has not been shown (34). Given the inherent inflammatory response of the trauma, there is a higher chance of false positives. Therefore, all data points should be considered in relation to the patient’s pretest probability. We found no evidence reported on the use of intraoperative ‘point-of-care’ PJI biomarker-associated diagnosis (35) in the context of PFFs, although it may be reasonable to be considered in selected cases. The use of synovial fluid markers of PJI should also be considered. D-lactate is a pathogen-specific marker, compared to alpha-defensin and leukocyte esterase produced by neutrophils (36). Consequently, D-lactate may be helpful in distinguishing inflammation from infection versus trauma. However, synovial fluid markers have not been specifically evaluated in the setting of fracture, and Fernando *et al.* recommended further studies to determine their clinical utility prior to widespread clinical application (31).

A recent systematic review found little evidence to guide treatment for patients with periprosthetic fractures where concomitant infection is confirmed or suspected (31). Several case series suggest that both a one-stage and two-stage exchange may be acceptable treatment strategies (37, 38). While one-stage treatment of PJI is favored in Europe and Asia and two-stage treatment remains the gold standard in North America (39), the decision to pursue one- versus two-stage exchange in the setting of PFFs must consider the physiologic reserve of a patient and whether he or she is able to undergo an extended multistage revision over several months (expert consensus). Both PFF and PJI carry significantly high rates of mortality, and 5-year mortality following PJI has been shown to surpass that of common cancers (40, 41). Thus, for patients with limited life expectancy, a one-stage exchange combined with long-term antibiotic suppression may be preferable. However, for healthier patients in whom the suspicion for infection is high, staged exchange with the goal of infection eradication may be possible.

**Figure 4:**
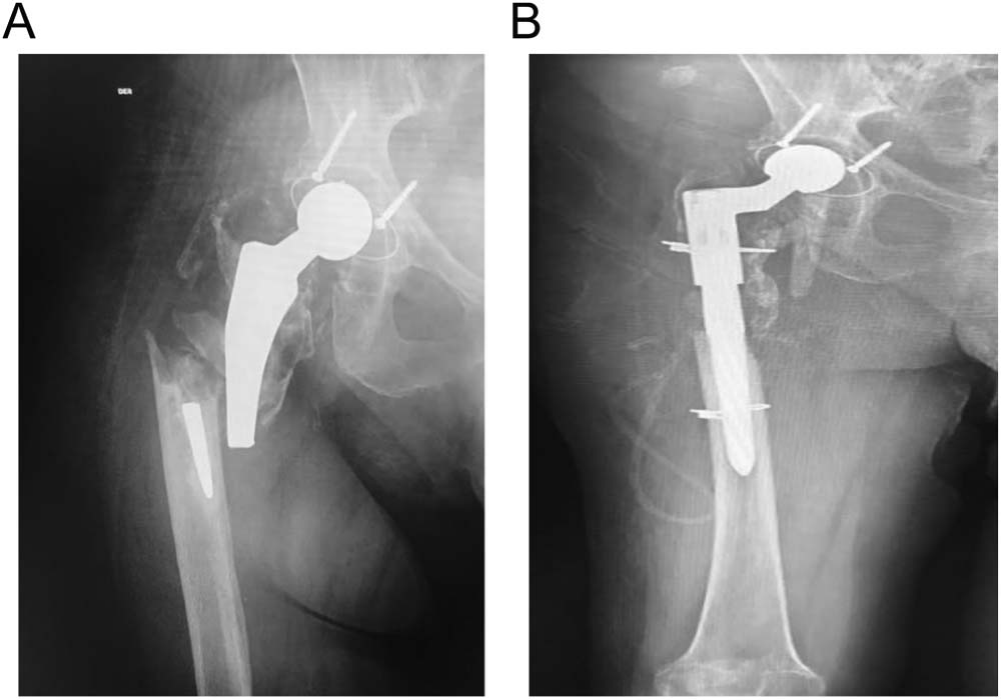
(A) Vancouver B3 fracture with catastrophic failure of the CS, with severe ectatic osteolytic defects, in a low-demand 88-year-old female. (B) Uncemented, fluted, modular, tapered stem revision of a B2L fracture, using Berry’s technique (69).

Aseptic osteolysis can also be a significant contributor to PFFs around CSs (Fig. 4A), in some cases precluding fixation and necessitating revision surgery (Fig. 4B). Osteolytic defects can be a cavitary cortical defect (loss of endosteal cortical bone without violation of the outer conical shell of the femur) or an ectatic defect (cavitary defect in which the femoral medullary canal is expanded) (42). CT scans can help evaluate the type, dimension, and location of the osteolytic lesions. Hozack’s technique (42) of uncemented revision, using bone allograft supplementation when necessary, may provide a reliable solution. Although acetabular revision or polyethylene exchange may be appropriate for PFFs with osteolysis, the decision must be weighed against the patient’s specific risk profile, since revising the acetabulum may increase surgical time and morbidity.

## Surgical techniques

The overarching goal when treating patients with PFFs around a CS is to achieve early mobilization to avoid the complications of prolonged bed rest. Treatment strategies to achieve this goal range from conservative management for patients with severely limited life expectancy, to osteosynthesis, to revision arthroplasty. Where there is supportive bone stock and a stable stem, osteosynthesis is preferred, sometimes with placement of a new CS (cement-in-cement technique) when the offset or anteversion in the existing implant needs to be revised. Revision arthroplasty is considered when there is a poor bone–cement interface, leading to a loose stem, non-supportive metaphyseal bone, or implant breakage. The following sections detail the considerations and technical pearls for each of these treatment strategies.

### Conservative treatment

While surgery is often required, some patients may not benefit from operative intervention. In fact, a review of management strategies for PFFs in the United Kingdom found that 23% of such injuries were managed without surgery (43).

After a formal process of shared decision-making, certain patients may be managed conservatively if their pre-morbid functional status makes postoperative mobilization unlikely (for example, in a patient who is non-ambulatory at baseline) or if their medical comorbidities or frailty puts them at an excessive risk of mortality. This type of management has been referred to as palliative care in hip fractures (44) and might be applicable in selected cases of PFFs.

In addition, some fracture patterns may be managed conservatively, including Vancouver A_G_ and A_L_ that are minimally displaced and not associated with instability. A period of limited weight-bearing and bracing may be sufficient (45, 46). More significant patterns around the femoral stem (Vancouver B) must not be mistaken for these rare trochanteric variants, as mobilization and successful union are unlikely with non-operative management of Vancouver B patterns.

### Osteosynthesis with retention of the original stem

Osteosynthesis of PFFs around CSs requires both the identification of a pattern that can be managed successfully with ORIF and the satisfactory execution using modern techniques.

For B1 patterns around CSs, ORIF is often preferred, but at least two exceptions exist. First, transverse and short oblique patterns at the tip of the femoral component have high failure rates with isolated ORIF and may benefit from revision arthroplasty (47). This may be due to the small bony surface area available for healing, from both the orientation of the fracture and the presence of cement, and the high strain environment in such patterns. Second, patients managed with ORIF for a B1 pattern presenting with failure of the index procedure may be best managed with revision rather than a second attempt at osteosynthesis (48).

For B2 patterns, some fractures benefit from isolated ORIF rather than revision arthroplasty. B2 fractures around CB stems may benefit from revision with or without ORIF (10). For B2 fractures around a PTS stem, recent studies have shown isolated ORIF not only to be a viable treatment option but also to have lower morbidity than revision arthroplasty (27, 28, 49). A multicenter study showed that among the critical advantages of ORIF over revision surgery when treating type B2 fractures around a PTS stem are the significantly lower rate of reoperations and the lower risk of transfusions and postoperative admission to the ICU. Moreover, the authors found that the 2-year survivorship after ORIF was 91% compared to 83% in the revision group (49).

Certain patterns, however, may not be appropriate for ORIF and instead require revision. These include comminuted metaphyseal split patterns, calcar fragments unable to be controlled with the fixation strategy, loosening of the bone–cement interface, and impacted PTS stems (50).

In addition, correct technical execution is important for obtaining good results. Anatomic reduction, rigid fixation that maximizes fixation opportunities around the implant, spanning of the entire bone with the construct, respect for the biology of the soft tissues and bone, and attention to the integrity of the cement mantle all contribute to a successful outcome. Non-anatomic reductions produce bridging constructs and do not allow the host bone to share the load during weight-bearing, adding considerable load to the implants and speeding their fatigue and failure. Non-anatomic reductions also do not reproduce the support of the index cement mantle for PTS designs and do not reestablish the environment for controlled subsidence required with these designs. Of note, anatomic reductions often necessitate an extensive, direct approach to the fracture site, which surgeons may wish to avoid in this frail population. However, all the benefits of a less invasive surgery are lost if a poor reduction results.

Along with an anatomic reduction goes rigid fixation. Cerclage of fracture fragments followed by stout locked plating to neutralize the compression is often required (expert consensus). Proximally around the stem, bicortical fixation should be sought where possible, and locking screws should be used liberally to gain axial and rotational stability. Additional cerclage over the plate and bone helps prevent the plate from pulling off the proximal segment. Overlap of the plate and implant of at least 6 cm, though ideally the entire proximal femur, has been shown to prevent failure (51). Distal to the implant, either standard or locking screws may be sufficient for adequate purchase.

While distal fixation is typically available in abundance, the extent of the fixation and how to apply it are worth considering. Spanning the entire femur may provide more fixation than is required to adequately fix into the distal segment, but it protects the distal bone stock from a stress riser at the end of a shorter plate and may reduce distal fracture risk. In addition, this portion of the fixation may proceed in a minimally invasive fashion, with the plate slid in a submuscular fashion and the screws placed percutaneously, adding little to the morbidity of the procedure in terms of dissection or time.

Respect for the soft tissues and prior cement mantle must also be kept in mind. While anatomic reduction may require a large exposure, this does not justify rough handling of the muscle or periosteum. Careful dissection to preserve these sources of blood supply is recommended. In addition, while fixation into a prior cement mantle can increase the purchase of individual screws, it is important to prevent cracking of the cement during screw insertion. Use of a slightly larger drill, while considering the screw’s core and outer diameters, may prevent cement mantle damage (52).

**Figure 5:**
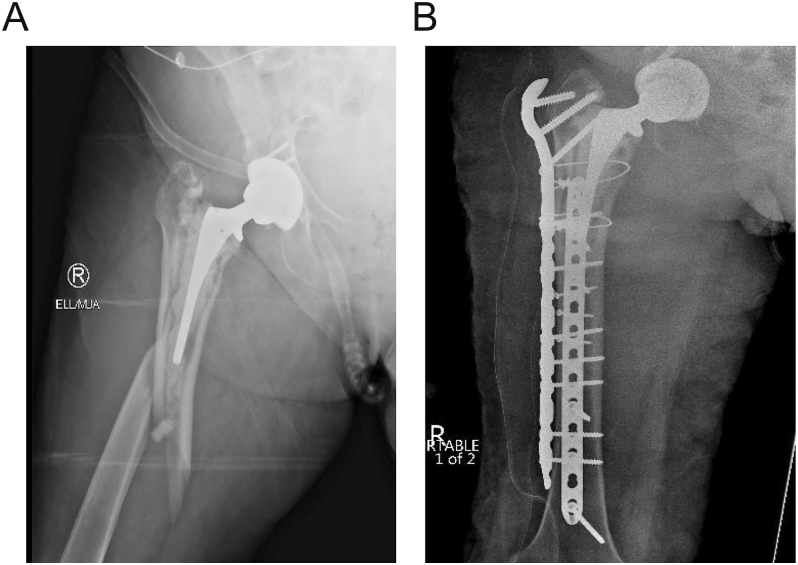
(A) Preoperative image of a low-demand 97-year-old female with a B2L fracture. (B) ORIF was performed with orthogonal plates, and immediate weight-bearing was allowed. The patient was a poor candidate for revision surgery due to a high-risk profile.

Finally, orthogonal fixation with dual plates or a plate and a strut graft may be helpful (expert consensus) (Fig. 5A and B). While clinical data remain minimal, biomechanical data show the expected increased strength of orthogonal fixation, and such constructs may encourage surgeons to allow earlier weight-bearing (53, 54, 55). Often an orthogonal plate can be applied to the anterior portion of the femur without additional dissection. If screw trajectory is a concern through an anterior plate, a femoral strut graft can also be cerclaged to augment laterally based fixation.

Data evaluating the use of femoral struts are of poor quality and quantity, although union rates and function outcomes have been encouraging in the series that have been published. Indications for strut grafts include young patients with Vancouver B2 or B3 fractures in which bone stock needs to be re-established (56). Strut grafting may also be considered in cases with transverse fractures at the tips of stems or revision open reduction internal fixation; however, as previously mentioned, both of these scenarios may be best managed with revision arthroplasty. Fresh frozen cortical strut allografts can be placed on the lateral, anterior, or medial aspect of the femur and cerclaged in place, while the plate is placed laterally (57). Morselized cancellous allograft can also be placed between the host femur and the allograft. In practice, due to improved outcomes with locked plating, the use of strut grafting has contracted, and we limit its use as an adjunct in osteosynthesis cases where healing times are expected to be prolonged (expert consensus). Some authors have also raised concerns that the soft tissue stripping necessary for the application of the strut graft may increase the rate of infection and risk of delayed union (58). Ultimately, if osteosynthesis is pursued, collaboration with an orthopedic trauma surgeon may be valuable.

### Osteosynthesis plus revising to a new cemented stem (cement-in-cement)

Occasionally, despite a cement–bone interface amenable to ORIF, maintaining the current CS can be detrimental. These scenarios include i) a stem with insufficient offset and high risk of dislocation, ii) a stem with inappropriate anteversion or leg length, and iii) a monoblock stem in a hemiarthroplasty. In these circumstances, in addition to performing ORIF, a cement-in-cement (CIC) technique may be helpful.

The CIC technique consists of the following steps (59): i) removal of the existing femoral stem from the intact cement mantle, ii) curettage of the proximal cement mantle to a depth where the cement can be visually confirmed as sound, and iii) roughening of the inner surface of the intact cement mantle with a high-speed burr. Subsequently, it is possible to choose either a short CS with a plate spanning the entire length of the femur (60) or a long CS bypassing the fracture site together with cerclage fixation of the fracture (22). For revision surgery involving a CIC technique, we recommend ample surgical exposure and a mechanically stable ORIF construct before implanting a new CS.

The CIC technique has been successful in various revision scenarios (61). Particularly in PFFs, CIC was described in a case series, although a high rate of reoperation was noted for causes other than stem fixation failure (62). A study by McCarthy *et al.* (63) reviewed the outcomes of 20 patients with CIC combined with ORIF for Vancouver B PFFs. Six cases (30%) required re-revision surgery, with four due to instability. Despite being a viable technique, CIC as part of the treatment strategy for PFFs remains rare. It accounted for 5.3% of the 22,170 revisions recorded in the Swedish Hip Arthroplasty Register as reported by Cnudde *et al.* (64). We recommend considering the CIC technique when managing a PFF around a PTS stem (expert consensus). Most likely, CIC is not ideal when managing fractures in CB stems that rely on a rigid cement–implant interface for stability.

One concern regarding this technique is the potential for new cement to be interposed at the fracture site, preventing adequate bone contact and healing. However, in a cadaveric study, Brew *et al.* demonstrated that cement interposition in the fracture is minimal (65).

### Revision to an uncemented tapered fluted titanium stem

The decision to revise a CS in a PFF is based on several factors: i) a poor bone–cement interface, including definitive evidence of loosening, whether found preoperatively or intraoperatively, ii) non-supportive bone stock in the metaphysis, iii) catastrophic failure of the stem (implant breakage), and iv) the inability to perform a cement-in-cement technique due to size or stem type incompatibility.

Revision surgery for PFFs is best performed by an arthroplasty surgeon and consists of two main approaches. The first is the use of a longer CS, potentially combined with impaction bone grafting techniques (66); the second is the use of an uncemented, fluted, tapered stem. The latter is the authors’ preferred technique (Fig. 2B).

Several considerations may be helpful to bear in mind for revision arthroplasty.

- Surgical approach: surgeons should choose the approach they are most familiar with. Observational evidence suggests that an approach different from the one used in the primary surgery does not affect the rate of complications (67, 68). Lateral decubitus positioning is our preferred approach as it facilitates lateral, posterior, transfemoral, or even ‘transfragmentary’ cement removal and expedites the use of intraoperative fluoroscopy (69). The posterior approach allows excellent leg mobilization to prepare the femoral canal. For shorter patients with an anteriorly bowed femur, the lateral positioning may help avoid anterior perforation. If varus remodeling is present (70), the lateral positioning also aids in performing a Wagner or Paprosky osteotomy if necessary (71).
- Personality of the fracture: we advocate for the use of CT scans in all cases of PFFs to understand the fracture pattern and develop strategies for fracture fixation (expert consensus). In addition, Hounsfield units (HU) from the CT scan can provide a surrogate measure of bone density and osteolysis, which can be helpful in decision-making (72). CT scans can also identify the location of the remaining cement within the femur; cement removal can be challenging and potentially lead to further complications in fractured bone with osteolysis. In our practice, we use the cement-splitting osteotomes and long stemmed curettes and hooks for cement removal.
- Anatomy of the femur and bone stock for femoral fixation: the CT scan can give information on diameter of the remaining femoral canal and the length of the isthmus, which is important for planning a fluted tapered revision stem. 4 cm of cortical bone–implant contact have been described as necessary to avoid subsidence, and 2 cm of contact are insufficient regardless the taper angle used (73). In addition, the shape and curvature of the femur may influence the length of the stem chosen. Full-length femur radiographs from both the affected side and the contralateral side are helpful in the preoperative evaluation. Accurate templating using digital software on both the affected and the contralateral side can assist in determining the best stem and size for the patient. In addition, intraoperative fluoroscopy is useful during implantation of a revision stem to avoid complications. We recommend placing at least one cable distal to the fracture before reaming to prevent fracture propagation (expert consensus). We also suggest on-power reaming to cortical chatter and obtaining at least 4 cm of diaphyseal engagement to avoid subsidence and loosening. In general, reaming to achieve a slightly larger diameter stem can help provide greater stability (expert consensus).
- Type of fluted uncemented stem: differences in design, shape, modularity, price, and clinical results help determine the optimal stem choice for a patient’s needs. To our knowledge, many authors have reported excellent results using fluted tapered stems in managing PFFs, but there are no studies comparing the performance of different stems (74, 75, 76, 77). We believe that data support the use of either modular or non-modular stems in PFFs (78, 79), and modularity should not be considered essential when managing these cases.
- Bearing surface and acetabular revision: several factors are considered when evaluating the need for acetabular revision during PFF surgery. i) Risk of dislocation and instability. If compromised greater trochanter fragments increase the risk of dislocation and instability by rendering the abductor mechanism insufficient, the surgeon may consider using a dual mobility construct (80), cementing a dual mobility liner in an existing cup (81), employing a focally constrained liner (82), or choosing a constrained liner (83), depending on the case. ii) Cup orientation and fixation. If the cup is not well positioned, the surgeon may consider cementing a liner or revising the cup. iii) Presence of osteolysis. If osteolysis contributed to the PFF, we recommend using a ceramic head instead of a cobalt chrome head.

### Proximal femoral replacement (PFR)

PFR should be a last resort in managing PFFs. Even in the most challenging scenarios, such as Vancouver B3 fractures, fluted, tapered uncemented stems have demonstrated success (69, 76), so the need to perform a PFR is exceptional.

The use of a PFR necessitates meticulous planning and execution, along with significant surgical expertise (84, 85). The inherent instability risk associated with these constructs often necessitates the use of constrained liners or dual mobility implants.

## Prognosis

### Decreasing short-term complications after surgery

PFFs most often occur in elderly, frail patients and necessitate major, urgent, unscheduled surgery, which is associated with significant perioperative morbidity and mortality; indeed, close to 5% of such patients may not survive the hospitalization (86).

Dislocation is a recognized complication after revision arthroplasty for any reason, including PFFs, with a 10% probability in this setting (87). If instability remains a concern despite proper component position, then dual mobility or constrained liners should be employed (87).

Surgical site infection is another known complication. Proper antibiotic prophylaxis, judicious use of blood transfusions, and early recognition remain the primary tools against this devastating problem.

Medical complications are frequent in this population. Pneumonias and urinary tract infections are common, with rates between 10 and 15% (87). Co-management with a geriatric or internal medicine team and reducing delays to the operating room are proven strategies to reduce these complications (87, 88). Proper use of DVT prophylaxis is also essential.

### Morbidity and risk of mortality

PFFs are associated with a high risk of mortality at one year, estimated at 11–18% (7, 40, 89, 90). Mortality rate at 1 year is greater in older patients, those with more medical comorbidities, and those previously in a dependent living situation (40). Boylan *et al.* showed that mortality rate following PFFs was equivalent to that of native femoral neck fractures at one month after surgery, accounting for age, sex, and comorbidity (91). However, mortality rates were significantly lower at 6 months and a year postoperatively. Unlike femoral neck fractures, which are associated with higher mortality for increased wait times for surgery (92), PFFs were not associated with a higher mortality rate with increased wait times (88), but Gibbs *et al.* demonstrated higher rates of medical complications with delays to operative management.

### Postoperative management and rehabilitation

The postoperative management of PFFs is not well studied. In a scoping review in 2024 investigating postoperative weight-bearing protocols, seven studies met inclusion criteria, which evaluated the outcomes of 22 patients (93). The non-weight-bearing group had no complications, the restricted weight-bearing group had one death and one implant failure, and the full weight-bearing group had one deep infection and one plate removal due to impingement. A survey by the same group found that postoperative weight-bearing recommendations varied widely among orthopedic surgeons in the Netherlands for a given fracture pattern and fixation method, although this study was limited by a low response rate and convenience sampling (94). No other studies exist to help guide optimal weight-bearing status following a PFF. Given the paucity of the literature on this subject, future studies should prioritize evaluating the impact of postoperative rehabilitation protocols on outcomes for patients with PFFs. Ultimately, we recommend that the treating surgeon determine the weight-bearing status considering the surgical construct, the patient’s activity level, and the patients’ comorbidities, with a goal to allow early weight-bearing whenever possible due to the complications associated with extended bed rest (expert consensus). In addition, limited evidence suggests that older patients may have greater difficulty complying with weight-bearing restrictions (95). Despite all efforts, functional outcomes remain suboptimal when treating PFFs (96).

## Conclusion

PFFs around CSs represent a complex scenario for an often-frail patient, with high rates of morbidity and mortality. We strongly advocate for the use of CT scans to assess the fracture pattern and the bone–cement and cement–implant interfaces, and we recommend evaluation for possible prosthetic joint infection, tailored to the patient’s pretest probability. Surgical decision-making and treatment strategies have evolved over time, with the primary goal to enable early mobilization. While non-operative treatment may be appropriate in select scenarios, most PFFs require surgical fixation. Depending on the fracture pattern and the status of the cement mantle and remaining bone stock, appropriate surgical management may comprise osteosynthesis, a cement-in-cement technique, or revision to a tapered fluted stem. Rarely, proximal femoral replacement may be required. Promising areas of future research include improving tools and diagnostic criteria to evaluate for PJI in the setting of PFFs and understanding how to optimize postoperative rehabilitation protocols to help reduce the high rates of medical and surgical complications associated with treating these difficult cases.

## ICMJE Statement of Interest

JWK has received the 2022 AFSH Resident and Fellows Fast Track Grant from the American Foundation for Surgery of the Hand; he has reveived travel grants from the American Association of Hip and Knee Surgeons, and Smith+Nephew. SAB has received grants (2022, 2023) from Google to perform sensor based research focused on human mobility. There is no direct or indirect relationship to Digital Health as this is basic science research. He has received royalties from Stryker related to the design of implants (hip and knee), and from Elsevier related to a surgical technique guide. He hold stocks in Archetypeai.io (a physical AI company that uses sensor data to understand the “real” world), GaitScience (manufacturer of nerve catheter for pain management), SiraMedical (company creating holograms from MRIs for surgical planning and education. SAB has received suture anchors for a biomechanical research study (donated through a research grant) from ConMed. All other authors declare that there is no conflict of interest that could be perceived as prejudicing the impartiality of the work reported.

## Funding Statement

This work did not receive any specific grant from any funding agency in the public, commercial, or not-for-profit sector.
